# A comprehensive bioinformatics analysis of FOXP3 in nonsmall cell lung cancer

**DOI:** 10.1097/MD.0000000000032102

**Published:** 2022-12-16

**Authors:** Jianfei Zhu, Zhenzhen Li, Jie Chen, Wensheng Li, Hongtao Wang, Tao Jiang, Yu Ma

**Affiliations:** a Department of Thoracic Surgery, Shaanxi Provincial People’s Hospital, Xi’an City, Shaanxi Province, China; b Department of Thoracic Surgery, Tangdu Hospital, Fourth Military Medical University, Xi’an City, Shaanxi Province, China; c Department of Pathology, Shaanxi Provincial People’s Hospital, Xi’an City, Shaanxi Province, China.

**Keywords:** FOXP3, nonsmall cell lung cancer, Tregs, tumor microenvironment

## Abstract

Fork head box p3 (FOXP3), the specific transcription factors of Tregs, not only in Tregs, but also expressed in cancer cells of certain malignant tumors. The histological positioning of FOXP3 in nonsmall cell lung cancer (NSCLC) and its biological significance are still unclear. This study aims to clarify the biological function of FOXP3 in NSCLC through bioinformatics analysis. Tumor immune estimation resource database was used to analyze the mRNA expression of FOXP3 in pan cancer, and to analyze the correlation between FOXP3 expression and tumor microenvironment cell infiltration. Overall survival and disease-free survival analyses were performed using a Kaplan–Meier plotter. Immunohistochemistry staining of FOXP3 was performed using human protein atalas (HPA) database, and immunofluorescence (IF) staining was used to verify gene expression and identify cell types. Protein–protein interaction (PPI) networks were drawn using STRING and visualized by Cytoscape. The functional and pathway enrichment analysis of FOXP3 used the DAVID database. In NSCLC, whether it is lung squamous cell carcinoma (*P* < .001) or lung adenocarcinoma (*P* < .001), FOXP3 is highly expressed in cancer tissue compared with normal tissue. Immunohistochemistry results showed that FOXP3 was mainly expressed in Tregs, but not in lung cancer tissues. IF staining showed that FOXP3 and CD3 (a marker of T cells) were co-expressed in immune cells. Moreover, survival analysis showed that high FOXP3 expression could be used as a predictor of poor overall survival (HR: 1.25, *P* = .00065) and disease-free survival (HR: 1.88, *P* = 1.1E-10) in patients with NSCLC. Next, we identified an important module containing 11 genes in the PPI network, including JUN, NFATC, STAT3, IRF4, IL2, IFGN, CTLA4, TNFRSF18, IL2A, KAT5, and FOXP3. KEGG signaling pathway was enriched in T cell receptor signaling pathway, Jak-STAT signaling pathway, cytokine–cytokine receptor interaction. Finally, we observed that FOXP3 expression correlated with infiltration of CD8 + T cells (*R* = 0.276, *P* = 5.90E−10), CD4 + T cells (*R* = 0.643, *P* = 6.81E−58), neutrophils (*R* = 0.525, *P* = 1.57E−35), and dendritic cells (*R* = 0.608, *P* = 1.35E−50) in lung adenocarcinoma, the same results were observed in lung squamous cell carcinoma. The infiltration of FOXP3-positive Tregs might promote the malignant progression of NSCLC, and targeted intervention of Tregs may be a potential treatment option for patients with NSCLC.

## 1. Introduction

In recent years, immunotherapy represented by immune checkpoint inhibitors has made significant breakthroughs in nonsmall cell lung cancer (NSCLC). Whether it is the first-line treatment of stage IV patients or the adjuvant/neoadjuvant treatment of early stage patients, amazing results have been achieved, opening a new era of NSCLC treatment.^[1–3]^ However, the existing immunotherapy models still face many challenges in NSCLC, such as treatment insensitivity, inevitable drug resistance, and how to obtain long-term survival benefits, all of which indicate that the current immunotherapy research for NSCLC is insufficient.^[4,5]^ The reason is that the existing immune escape theory and its clinical application focus on a single immune checkpoint corresponding to T cells in the adaptive immune system, and it is difficult to cope with the extensive heterogeneity and complex carcinogenic mechanism of NSCLC.^[6]^ Therefore, new immune escape theories and immunotherapy methods need to be explored and applied urgently.

Fork head box p3 (FOXP3), a signature transcription factor of Tregs, was mainly specifically expressed in Tregs. Infiltration of FOXP3 + Treg cells can induce immunosuppressive function of tumor microenvironment and shorten survival of patients with NSCLC.^[7,8]^ However, several studies reported that FOXP3 can be highly expressed in ovarian, and breast cancer tumor cells and corresponding Tregs at the same time.^[9,10]^ A study on pancreatic cancer confirmed that FOXP3 promotes pancreatic cancer progression by directly transactivating CCL5 to recruit Tregs to infiltrate.^[11]^ The role of FOXP3 in tumor cells and TME cells of NSCLC is still being explored.

Based on the above studies, we proposed the following scientific hypothesis that FOXP3 specifically activates chemokines, thereby promoting the infiltration of Tregs, and finally promoting the infiltration and metastasis of NSCLC. This study aimed to comprehensively study the biological role of FOXP3 in NSCLC through bioinformatics analysis, and provide new targets for the diagnosis and treatment of NSCLC.

## 2. Methods and materials

### 2.1. TIMER (tumor immune estimation resource) database

DiffExp module of the TIMER database^[12]^ (https://cistrome.shinyapps.io/timer/) was used to study the differential expression of target genes between tumors and paired normal tissues. Statistical significance of differential expression was assessed using the Wilcoxon test. Gene module was used to correlate the expression of a gene of interest with immune cell infiltration. Immune cells mainly include the following 6 types of cells: B cells, CD8 + T cells, CD4 + T cells, macrophages, neutrophils, and dendritic cells. *P* < .05 was considered statistically significant.

### 2.2. HPA database

HPA database was used to present the immunohistochemical (IHC) results of the gene of interest, and the typical results of IHC from 144 different individuals and 216 tumor tissues were in this database. The main purpose was to map the location of proteins encoded by expressed genes in human tissues and cells.

### 2.3. IF staining

After antigen retrieval and blocking, tissue sections were incubated with specific primary antibodies (EPCAM, Servicebio, GB14078, 1:200; FOXP3, Servicebio, GB11093, 1:200; CD3, Servicebio, GB11014, 1:200), which were mixed with horseradish oxidase-labeled secondary antibodies or fluorophore-labeled secondary antibodies were incubated and finally stained with diaminobenzidine and counterstained with hematoxylin. This study was approved by the ethics committee of the Shaanxi Provincial People’s Hospital (N0.20220424).

### 2.4. Kaplan–Meier plotter database

Kaplan–Meier plotter database^[13]^ (https://www.kmplot.com) was used to analyze the relationship between the expression level of FOXP3 and the prognosis of patients with NSCLC. Disease-free survival (DFS) and overall survival (OS) were mainly analyzed. All patients were divided into 2 groups according to the “automatic selection of the best critical value” expression (high expression and low expression), the Kaplan–Meier method was used to draw the survival curve, and *P* < .05 was considered statistically significant.

### 2.5. Constructing a PPI network

To analyze the FOXP3-related proteins in NSCLC, the STRING database^[14]^ (http://string-db.org) was used to draw a FOXP3-related protein network dendrogram, using Cytoscape software exported the visual network interaction map, and screened FOXP3-related proteins for subsequent gene ontology (GO) and kyoto encyclopedia of genes and genomes (KEGG) analysis.

### 2.6. Database for annotation, visualization, and integrated discovery (DAVID)

Functional and pathway enrichment analysis of FOXP3 in NSCLC by DAVID database^[15]^ (http://david.abcc.ncifcrf.gov/). GO enrichment analysis was performed on FOXP3 and the previously screened related genes, including biological process, cellular component and molecular function analysis. Moreover, KEGG was used to analyze the signaling pathway of FOXP3 and related genes.

## 3. Results

### 3.1. Expression levels of FOXP3 in NSCLC

**Figure 1.**
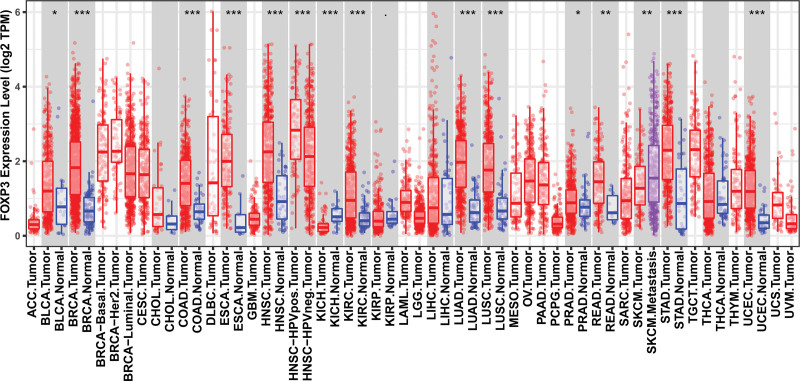
Differential expression of FOXP3 in tumor and normal tissues in pan cancer with TIMER database. FOXP3 = Fork head box p3, TIMER = tumor immune estimation resource.

**Figure 2.**
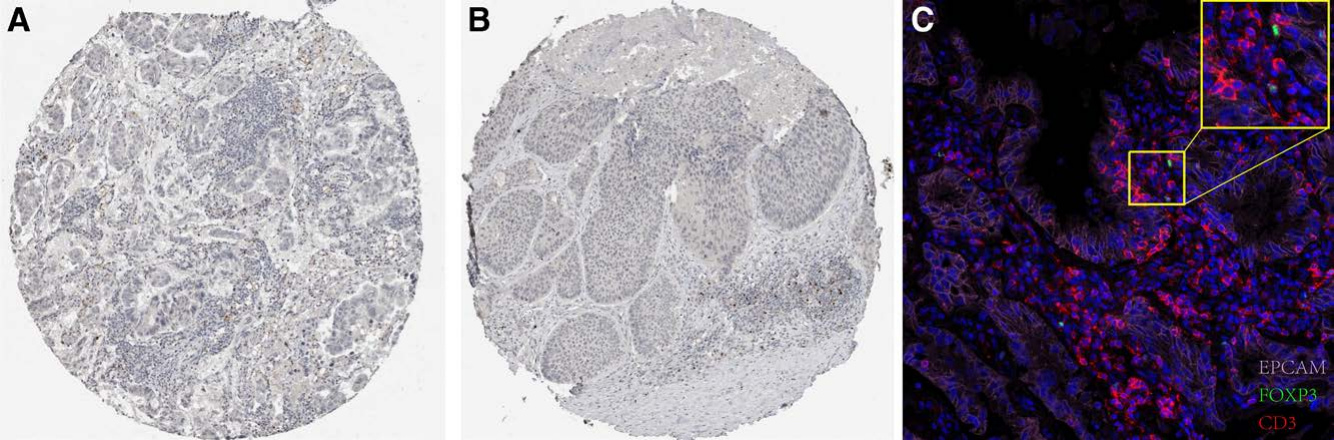
Verification of the protein expression of FOXP3 and cell type identification in NSCLC. IHC confirmed FOXP3 expression in T cells of lung adenocarcinoma (A), and lung squamous cell carcinoma (B); (C) IF staining verified the localization of FOXP3 and CD3 (T cell marker gene) and EPCAM (epithelial cell marker gene). FOXP3 = Fork head box p3, IHC = immunohistochemistry, IF = immunofluorescence, NSCLC = nonsmall cell lung cancer.

In the DiffExp module of the TIMER database, we observed the mRNA expression of FOXP3 gene in various common tumors. As shown in Fig. 1, compared with normal tissues, FOXP3 is highly expressed in a variety of tumors, including stomach cancer, esophageal cancer, and breast cancer and so on. It was worth noting that FOXP3 is highly expressed in lung squamous cell carcinoma (*P* < .001) and lung adenocarcinoma (*P* < .001) compared with normal tissues, suggesting that FOXP3 plays an oncogene role in pan cancer. Next, we observed the protein expression of FOXP3 gene in the HPA database. As shown in Figs. 2A and 2B, FOXP3 was not expressed in cancer cells, but mainly in immune cells. In IF staining, we verified that FOXP3 colocalized with the T cell marker gene CD3 in immune cells, but not colocalized with the epithelial marker gene EPCAM (Fig. 2C).

### 3.2. The prognostic predictive role of FOXP3 in NSCLC

**Figure 3.**
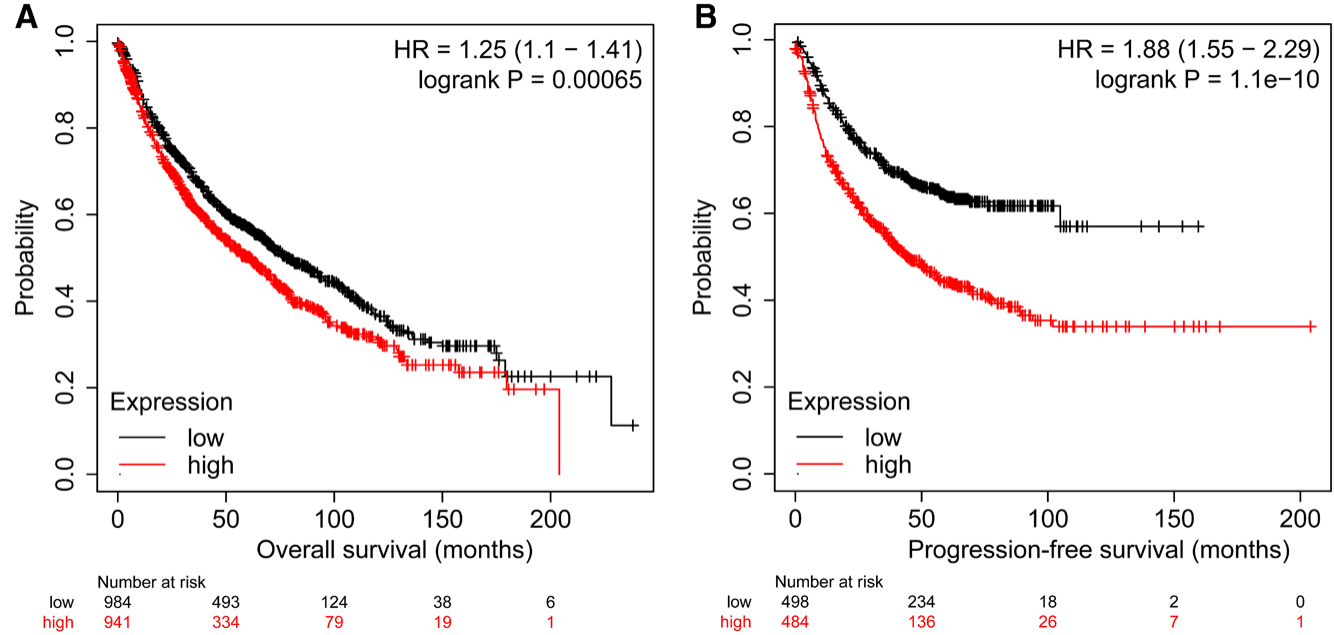
Survival curves of patients with NSCLC stratified by FOXP3 expression. (A) OS curves of patients with NSCLC according to FOXP3 expression; (B) DFS curves of patients with NSCLC according to FOXP3 expression. DFS = disease-free survival, FOXP3 = Fork head box p3, NSCLC = nonsmall cell lung cancer.

Survival analysis of 1925 patients with NSCLC from the Kaplan–Meier Plotter database, with 941 patients with high expression of FOXP3 and 984 patients with low expression of FOXP3. OS analysis shows that low-expression FOXP3 patients had a median OS of 78 months, which was significantly higher than those of high-expression FOXP3 patients (61.21 mo), *P* = .00065 (Fig. 3A). At the same time, DFS analysis was performed on 982 patients with early stage NSCLC, and the FOXP3 could be used as an indicator of DFS prediction (HR: 1.88, *P* = 1.1E−10), in Fig. 3B.

### 3.3. Interaction of FOXP3 with related proteins

**Figure 4.**
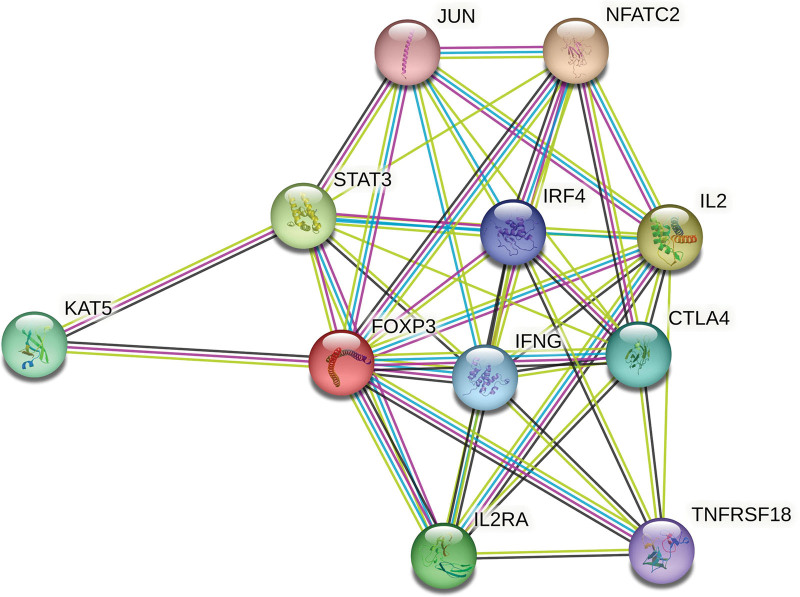
The network of FOXP3 and 10 proteins that significantly interacted with FOXP3 in NSCLC with STRING database. FOXP3 = Fork head box p3, NSCLC = nonsmall cell lung cancer.

Next, in order to better understand the functional mechanism of FOXP3, the STRING database was used to construct a PPI network of FOXP3. We observed 10 functional proteins have interactions with FOXP3 (Fig. 4), including JUN, NFATC, STAT3, IRF4, IL2, IFGN, CTLA4, TNFRSF18, IL2A, and KAT5. The more connectors between proteins, the stronger the relationship. Among the 10 predicted proteins identified above, 3 related proteins proved to be related to the development and prognosis of NSCLC.^[16,17]^

### 3.4. Gene enrichment and pathway functional annotation of FOXP3 in NSCLC

**Figure 5.**
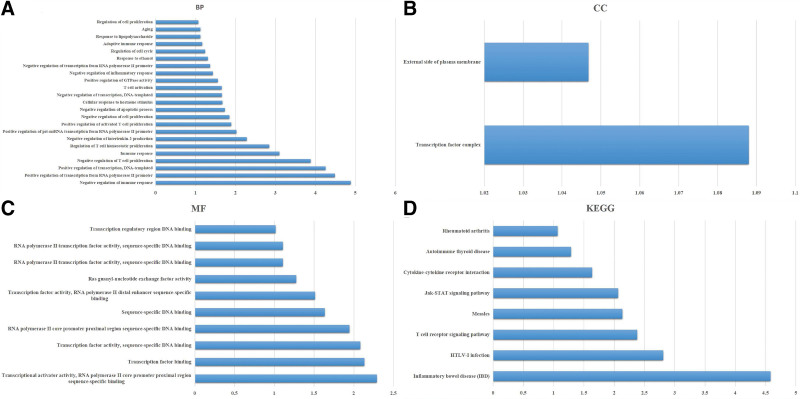
Functional enrichment analysis of FOXP3 in NSCLC. (A) GO-Biological Process, (B) GO-cellular component, and GO-molecular function (C) analyses to assess biological enrichment in NSCLC with high FOXP3 expression by GSEA; (D) KEGG pathway analysis to assess biological enrichment in NSCLC with high FOXP3 expression. FOXP3 = Fork head box p3, NSCLC = nonsmall cell lung cancer.

To learn about the features identified, we uploaded these 11 genes to the DAVID database to determine the GO classification and KEGG pathways. In the biological process, these genes mainly affect the immune response and T cell activation of NSCLC. As for molecular functions, these genes were mainly engaged in transcriptional active factors activation and transcription factors. In addition, the results of the cell component analysis showed that these gene sets can be enriched in transcription factor complex and external side of plasma membrane. Finally, KEGG signaling pathway was mainly enriched in the T-cell receptor signaling pathway, the Jak-STAT signaling pathway, and the cytokine–cytokine receptor interaction (Fig. 5).

### 3.5. Correlation between FOXP3 expression and immune cell infiltration in NSCLC

**Figure 6.**
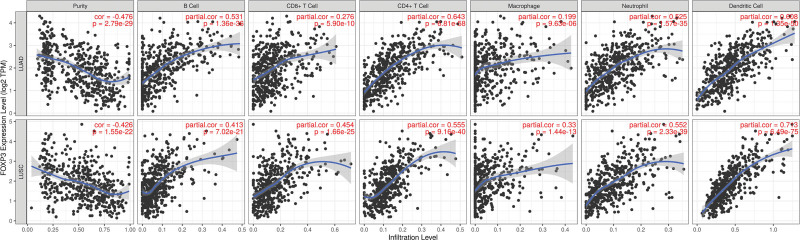
Correlation between FOXP3 expression and immune cell infiltration in LUAD (upper) and in LUSC (lower). FOXP3 = Fork head box p3, LUAD = lung adenocarcinoma, LUSC = lung squamous cell carcinoma.

The correlation between FOXP3 expression and immune cells is also one of our research goals. The TIMER database analysis was obtained, in lung adenocarcinoma, the expression of FOXP3 was inversely correlated with the purity of the tumor (r = −0.476, *P* = 2.79E−29); FOXP3 expression was positively correlated with the infiltration of common humoral immunity B cells (*R* = 0.531, *P* = 1.36E−36). Moreover, FOXP3 expression correlated with infiltration of CD8 + T cells (*R* = 0.276, *P* = 5.90E−10), CD4 + T cells (*R* = 0.643, *P* = 6.81E−58), neutrophils (*R* = 0.525, *P* = 1.57E−35), and dendritic cells (*R* = 0.608, *P* = 1.35E−50). We also found that FOXP3 expression is not obvious with macrophage infiltration in a TME (*R* = 0.199, *P* = 9.63E−06), the same results were found in lung squamous cell carcinoma (Fig. 6).

## 4. Discussion

As a characteristic transcription factor of Tregs, FOXP3 participates in the immune surveillance function of the body by promoting the differentiation of T cells into Tregs. However, several recent studies have shown that in addition to Tregs, FOXP3 has also been found to be expressed in various tumor cells, such as gastric cancer cells^[18]^ and renal cancer cells^[19]^. In this study, we comprehensively demonstrated the biological function of FOXP3 in NSCLC from mRNA, protein levels, and protein interactions through bioinformatics analysis.

The role of FOXP3 in malignant tumor cells is currently controversial. In breast cancer, FOXP3 can bind to the promoter regions of HER2 and SKP2, and further inhibit the expression of oncogenes HER2 and SKP2, ultimately inhibiting cell proliferation and promoting cell apoptosis.^[20]^ Moreover, the expression of FOXP3 can promote the transcription and translation of tumor suppressor genes p21 and LATS2 (p21 can specifically arrest the cell cycle in G1 phase, and overexpression of LATS2 can promote the degradation of the proliferation index YAP).^[21]^ The above findings suggest that FOXP3 functions as a tumor suppressor gene, and the same results have been observed in ovarian and prostate cancers.^[22,23]^ In contrast, in pancreatic tumors, 13 malignant adenocarcinoma specimens showed high FOXP3 expression, while 7 nonmalignant tumors also showed high FOXP3 expression. In addition, IHC staining showed that 24 of the 39 pancreatic cancer patients showed high expression of tumor FOXP3, and these FOXP3 staining was mostly localized in the cytoplasm, while only a few samples were localized in the nucleus. At the same time, this study showed that normal pancreatic ductal epithelial cells do not express FOXP3,^[24]^ suggesting that the interaction between FOXP3 + tumor cells and FOXP3 + positive Treg cells may promote the development of pancreatic cancer.

FOXP3 + Treg cells infiltration in TME of NSCLC is a focus of research. Previous study^[25]^ demonstrated that FOXP3 + Treg cell infiltration in tumor islets predicted a poor prognosis in NSCLC (HR: 3.91, *P* < .001). In N0 stage patients with NSCLC, tumor-infiltrating FOXP3 + Treg was positively correlated with intratumor COX-2 expression and with poor RFS, suggesting that FOXP3 + Treg cell infiltration was a predictor of poor prognosis.^[26]^ Additional studies have demonstrated that FOXP3 promotes tumor growth and metastasis in tumor cells in NSCLC by activating the Wnt/β-catenin signaling pathway and EMT.^[27,28]^ It is worth noting that in our study, we confirmed that FOXP3 is highly expressed in NSCLC, and the high expression of FOXP3 indicates that patients had poor DFS and OS, suggesting that FOXP3 plays a role in promoting cancer in NSCLC. However, the findings of IHC from HPA database showed that FOXP3 was mainly expressed in immune cells, and its expression was not observed in cancer cells. In addition, protein interaction and KEGG signaling pathway analysis suggest that FOXP3 plays a major role in NSCLC by mediating CTLA4 and T cell receptor related pathways, which is consistent with previous reports.^[29,30]^

Although our data comes from bioinformatics analysis of multiple databases, this study inevitably has certain shortcomings. First, the data comes from different databases, which is not convenient for integrated analysis; Second, advanced detection methods such as single-cell RNA sequencing (scRNA-seq) should be applied to our subsequent cell type identification of cancer cells and immune cells; Finally, the relevant findings should be verified by in vitro and in vivo experiments.

In summary, we comprehensively confirmed the high expression of FOXP3 in NSCLC and its predictive effect on prognosis using public databases. FOXP3 was mainly expressed in Tregs and plays a role in promoting cancer, suggesting that the infiltration of FOXP3-positive Tregs promotes the occurrence and development of NSCLC and provides a new target for its immunotherapy.

## Author contributions

Tao Jiang, Yu Ma, and Jianfei Zhu participated in study design and study conception; Jianfei Zhu and Yu Ma performed bioinformatics analysis; Tao Jiang, Hongtao Wang, and Jianfei Zhu performed the surgery; Yu Ma, Zhenzhen Li, Jie Chen, and Wensheng Li performed pathological diagnosis; Jianfei Zhu and Yu Ma recruited patients. Jianfei Zhu and Yu Ma drafted the manuscript. All authors provided critical review of the manuscript and approved the final draft for publication.

**Conceptualization:** Yu Ma.

**Data curation:** Hongtao Wang.

**Validation:** Zhenzhen Li, Jie Chen, Wensheng Ji.

**Writing—original draft:** Jianfei Zhu.

**Writing—review and editing:** Tao Jiang.
